# The role of hormones and aromatase inhibitors on breast tumor growth and general health in a postmenopausal mouse model

**DOI:** 10.1186/1477-7827-12-66

**Published:** 2014-07-15

**Authors:** Arunkumar Arumugam, Elaine A Lissner, Rajkumar Lakshmanaswamy

**Affiliations:** 1 Center of Excellence in Cancer Research, Department of Biomedical Sciences, Paul L. Foster School of Medicine, Texas Tech University Health Sciences Center, El Paso, Texas 79905, USA; 2 Parsemus Foundation, Berkeley, California 94702, USA

**Keywords:** Postmenopausal breast cancer, Aromatase inhibitors, Hormones, Bone markers, Cardiac markers

## Abstract

**Background:**

Breast cancer is the most frequently diagnosed cancer in women in the United States. Approximately 70% of breast cancers are diagnosed in postmenopausal women. Major clinical trials and experimental studies showed that aromatase inhibitors are effective against postmenopausal breast cancer. Despite their effectiveness in reducing tumor recurrence, aromatase inhibitors have adverse effects on the cardiovascular system and increase osteoporosis and bone fractures. Our study is aimed at investigating the role of natural steroid hormones on serum cardiovascular and bone resorption markers in an established mouse model mimicking postmenopausal breast cancer.

**Methods:**

Ovariectomized nude mice were transplanted with MCF-7 breast cancer cells constitutively expressing aromatase. The mice were treated with different combinations and doses of steroids, [estrogen (25 pg, 40 pg, 100 pg), progesterone (6 ng) and testosterone (50 ng)] along with dehydroepiandrostenedione (100 ug). Serum levels of HDL, LDL/VLDL, free and total cholesterol, total and bone specific alkaline phosphatase and triglycerides were analyzed after 5, 10 and 15 months.

**Results:**

Free cholesterol and LDL/VLDL levels in serum were reduced in groups mimicking estrous cycle and menstrual cycle hormones treatment. HDL cholesterol was increased in all the hormone treated groups except the estrous cycle-mimicking group. Bone specific alkaline phosphatase was decreased in menstrual cycle levels of estrogen and progesterone treatment.

**Conclusions:**

All together our results show that use of natural hormones in appropriate combinations have beneficial effects on cardiac and bone toxicity, along with better tumor reduction than current treatments.

## Background

Breast cancer is one of the most common cancers among women, with more than one million cases and nearly 600,000 deaths annually worldwide [1]. Breast cancer incidence rates vary markedly among countries. Breast cancer is the most frequently diagnosed cancer in women in the United States. Due to the high incidence rate along with social and cultural considerations, breast cancer ranks highest among women’s health concerns. Despite the advancement of new preventive strategies, the incidence of breast cancer has remained the same since 2005 [2]. Approximately 70% of breast cancers are diagnosed in postmenopausal women [3].

The steroid hormones estrogen and progesterone have long been thought to play a role in the etiology of breast cancer. Apart from breast cancer growth, these hormones also influence various physiological processes. After the cessation of ovarian function, a significant decrease in the ovarian hormones estrogen and progesterone leads to a variety of symptoms known as postmenopausal symptoms. The most common symptoms include hot flashes, night sweats, mood swings, and sleep disturbances. These symptoms negatively influence a woman’s quality of life. Additionally, estrogens have beneficial actions on bone and lipid metabolism and cardiovascular function [4-7]. To alleviate postmenopausal symptoms, hormone replacement therapy (HRT) is used as a treatment. In particular, HRT has been shown to alleviate vasomotor symptoms, aid in the prevention of osteoporosis and improve serum lipid profiles [8-11].

Despite positive effects of HRT, some exogenous hormones have been shown to increase the incidence of breast cancer. The Women’s Health Initiative (WHI) study, which utilized conjugated equine estrogen (0.625 mg per day) and medroxyprogesterone acetate (2.5 mg per day), revealed a 24% increased risk for invasive breast cancer [12], with no major beneficial effects against cardiovascular disease, stroke, and thromboembolic diseases [13]. These findings resulted in a 63% reduction of HRT use within 3 months after the WHI publication. However, recent analyses of the WHI data have shown that estrogen replacement therapy alone (without medroxyprogesterone acetate) actually decreased the risk of breast cancer [12].

Aromatase inhibitors (AIs) are widely used for the adjuvant treatment of postmenopausal breast cancer, generally prescribed for five years at the conclusion of surgery, chemotherapy and/or radiation treatment. AIs target the aromatase enzyme, which converts adrenal androgens to estrogens. After the Arimidex, Tamoxifen, Alone or in Combination (ATAC) trial showed AIs are equally effective to tamoxifen, the FDA approved AIs as a first-line endocrine therapy for preventing recurrence of hormone-positive postmenopausal breast cancer [14-18]. However, several observational and meta-analyses revealed that AIs used for the prevention of postmenopausal breast cancer reduce cancer recurrence but also have serious side effects on bone and the cardiovascular system. AIs cause severe joint pain, hip fracture, increased osteoporosis risk, and musculoskeletal pain. Loss of learning and memory function is also an important adverse effect associated with AI treatment that can lead to dementia at later stages [19,20]. In a large cohort study using 8,769 breast cancer patients, approximately 51% of the patients discontinued their adjuvant hormonal therapies including tamoxifen and AIs due to the adverse side effects [21]. Therefore, it is imperative to find alternative treatment regimens with fewer unfavorable side effects for postmenopausal breast cancer patients.

From the available literature and published data, it is clear that currently used treatments reduce breast cancer recurrence but also have serious undesirable side effects that limit their usefulness. In this study, we aimed to develop hormone treatments that will provide similar or improved survival rates compared with the drugs used currently, but without the harmful and undesirable side effects.

## Methods

### Animals

Female athymic nude mice were obtained from Harlan Laboratories™ (San Diego, CA). Animals were housed in groups in a pathogen-free environment under controlled light and humidity conditions, and received food and water *ad libitum*. The mice were ovariectomized at 10 weeks of age. One week later, mice received transplants of MCF-7 cells stably transfected with the human aromatase gene. Each experimental group had 15 animals and when necessary for validation, experiments were repeated. All procedures followed the Animal Care and Use Committee guidelines of Texas Tech University Health Sciences Center.

### Cell culture and xenograft transplantation

MCF-7 cells stably transfected with the human placental aromatase gene (MCF-7-ARO) were cultured in Eagle’s minimum essential media containing 10% fetal bovine serum and antibiotics. Subconfluent MCF-7-ARO cells were trypsinized and suspended in collagen matrix solution (85% collagen and 15% neutralizing buffer) to make a concentration of 3 10^7^ cells/ml. At 11 weeks of age, ovariectomized mice were inoculated with MCF-7-ARO cells. Each mouse was inoculated with 0.1 ml cell suspension in both flanks (~3 10^6^ cells/site). Tumor growth was determined by measuring tumor volume using the formula 4/3 r_1_^2^r_2_, where r_1_ is the minor radius and r_2_ is the major radius.

### Hormone treatments

**Figure 1:**
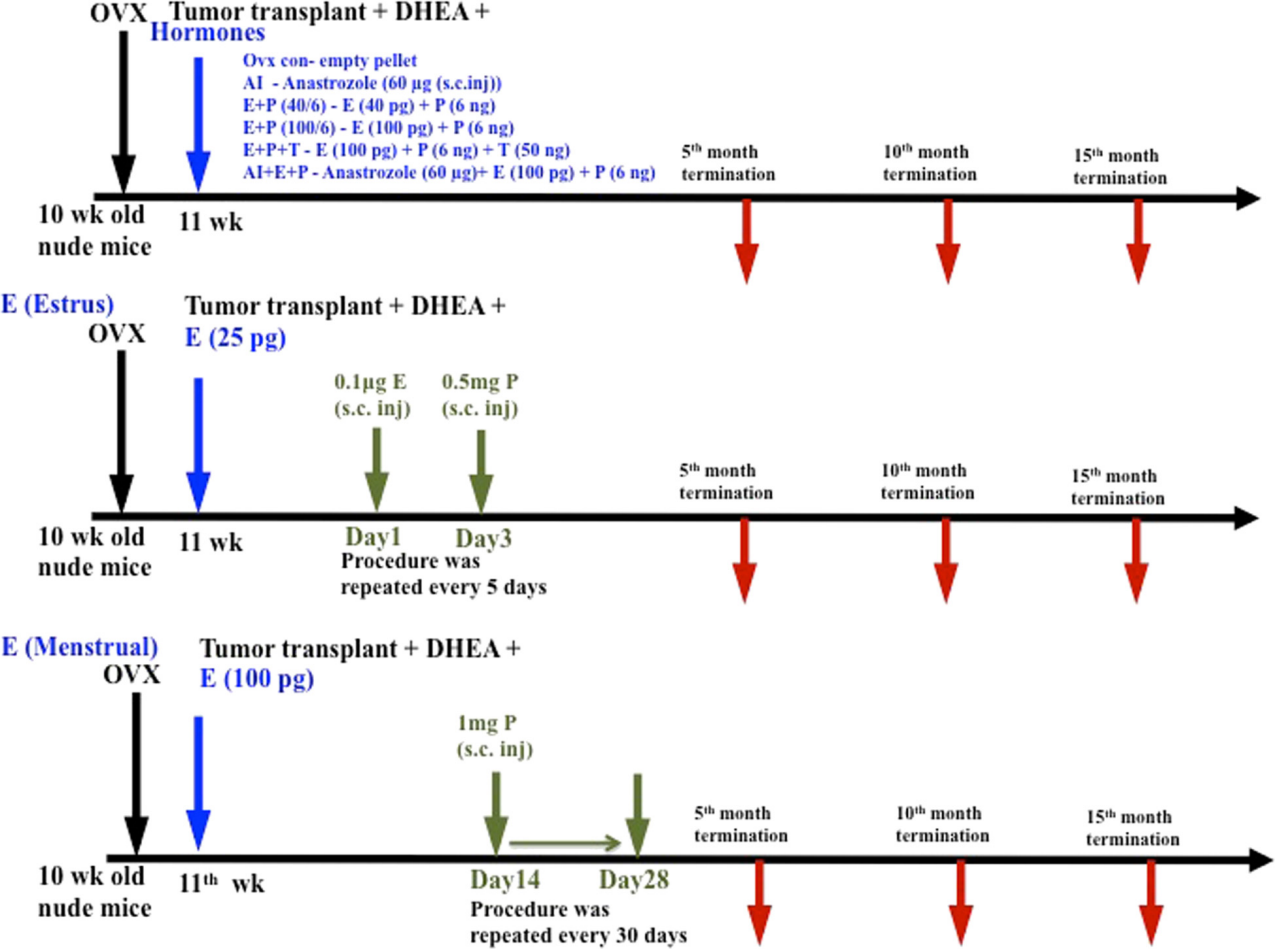
**Schematic representation of experimental setup.** One hundred and twenty female, nude mice were ovariectomized at 10 weeks of age. After a week MCF7-ARO cells were injected into the flanks (~3 × 10^6^ cells/site) and the animals were separated into 8 groups (n = 15). All the animals received 0.1 mg DHEA as daily subcutaneous injection. Ovariectomized control group animals did not receive any additional treatment, AI group animals received 60 μg anastrozole as subcutaneous injection for 7 weeks, E + P (40/6) group animals were implanted with E and P pellets that results in 40 pg E and 6 ng of P in circulation, E + P (100/6) delivers E and P to achieve 100 pg E and 6 ng P in circulation, E + P + T group animals received T pellets along with E and P pellets to achieve serum concentration of E-100 pg, P-6 ng and T-50 ng. AI + E + P group animals received 60 μg anastrozole as subcutaneous injection, for 7 weeks and E and P pellets to release E-100 pg and P-6 ng in circulation. Estrous group animals received E pellets releasing 25 pg of E in circulation and additionally they received 0.1 μg of E at day 1 and 0.5 mg of P at day 3 as a subcutaneous injection, to mimic estrus cycle. The estrus cycle treatment was repeated every 5 days, till the end of the experiment. Menstrual group animals were implanted with E pellets releasing 100 pg of E in circulation and 1 mg of P as a subcutaneous injection at day 14 through day 28 and the procedure was repeated every month to mimic human menstrual cycle. A set of 3 mice per group was terminated at 5, 10 and 15-month time points and remaining 6 animals were observed until ~30 months. A survival surgery was performed to remove tumors if they reach 500 mm^3^ in size.

Ten-week-old mice were ovariectomized and randomly separated into 8 groups consisting of 15 animals per group. Mice in all groups were inoculated with MCF-7-ARO cells at 11 weeks of age. As shown in Figure 1, the mice were either exposed to hormones continuously, or in a treatment mimicking the estrous cycle in the mouse (because we were using a mouse model) or human (because the implanted tumor tissue was of human origin). Throughout the experiments, ovariectomized animals received 0.1 mg dehydroepiandrosterone (DHEA) daily via subcutaneous injection, to allow the aromatization process which is responsible for much of postmenopausal hormone production and which is attacked by aromatase inhibitors. Estrogen (E), progesterone (P), and testosterone (T) were packed in individual silastic tubes. Dosages were adjusted such that they would result in 40 or 100 pg/ml estradiol, 6 ng/ml progesterone, and 50 ng/ml testosterone in circulation. Anastrozole, an aromatase inhibitor, which is used as an adjuvant therapy in postmenopausal breast cancers, was administered via subcutaneous injections (60 μg daily/mouse).

### Running wheel experiments

Mice were housed in groups of 3 animals per cage. A running wheel was placed in the cage of experimental animals to assess voluntary wheel running behavior as a measure of physical activity. The number of revolutions was monitored using a sensor connected to a computer. Because 3 mice were housed per cage, the average number of rotations per hour was calculated based on the total revolutions per hour divided by the number of mice in the cage.

### Morris water maze tests

The Morris water maze was used to measure cognition and the spatial learning ability of the animals [22]. Briefly, the water maze was a circular pool (120 cm in diameter, 40 cm in height) with water filled to 2 cm and maintained at a temperature of 20 ± 2C. A plastic square platform, 14 cm 14 cm, was placed 1 cm below the water level. Each mouse received five training trials (50 seconds each) for 7 consecutive days. During the first 2 days, we used a visible platform, but we used a hidden platform for all other days. Latency to escape from the water maze (the time to find the submerged platform) was calculated for each trial within the 50-second period. Swimming distance and speed were also analyzed. The percentage of mice that reached the platform in each group was calculated.

### Serum lipid and lipoprotein analyses

Animals were euthanized at different time points and serum was separated from whole blood collected and used for biochemical analyses. Serum levels of total cholesterol, free cholesterol, high-density lipoprotein (HDL), low-density lipoprotein (LDL), very-low-density lipoprotein (VLDL), and triglycerides were measured using commercially available kits (Biovision, Milpitas, CA).

### Total and bone-specific alkaline phosphatase analyses

Determination of serum alkaline phosphatase (ALP) levels reflects the bone health of animals [23]. ALP enzymatic activity was quantified using a p-nitrophenylphosphate (pNPP) colorimetric assay kit (Biovision, Milpitas, CA). Serum bone-specific alkaline phosphatase levels were determined using an ELISA kit (Cosmo Bio, Carlsbad, CA).

### Statistical analysis

The data are expressed as mean ± SEM. The Mann–Whitney test or Student’s t test was used to analyze differences between the groups using the GraphPad Prism 6 software package. Any value that was *P* < 0.05 was considered statistically significant.

## Results

### Effect of the aromatase inhibitor and hormones on mammary tumor growth

**Figure 2:**
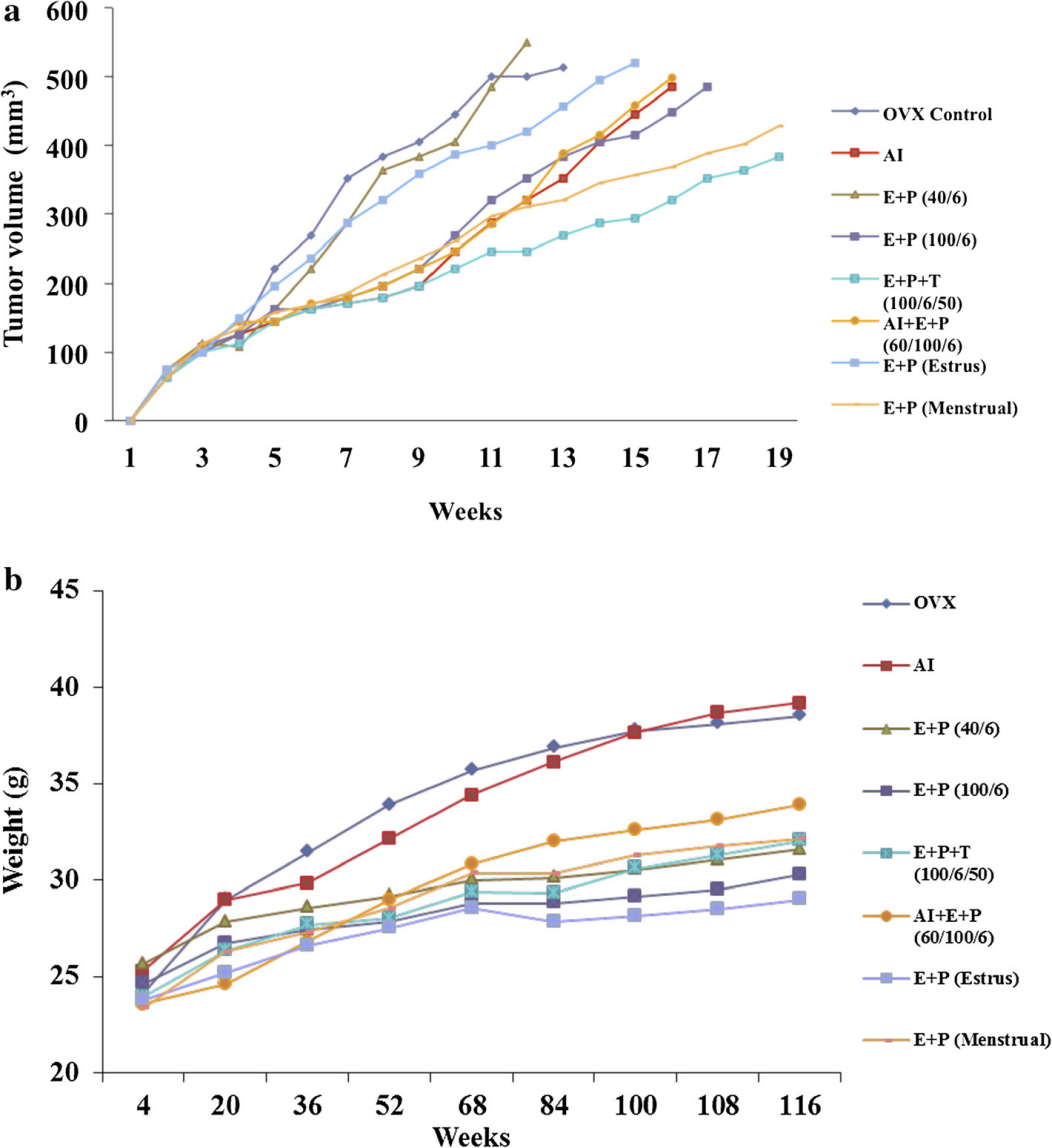
**Effect of hormones on MCF7-ARO tumor xenograft growth and body weight in female nude mice. a)** Mice were treated with AI and hormones according to the experimental setup described in Figure 1 in Methods. Tumor growth was measured twice a week and tumor volume was calculated using the formula 4/3 r_1_^2^r_2_, where r_1_ is the minor radius and r_2_ is the major radius. Tumor growth was rapid in ovariectomized control and mice received E + P (40/6), whereas E + P + T (100/6/50) and E + P (Menstrual) treatment reduced the tumor growth drastically. **b)** Body weight of the animals shows toxicity of the treatment and general health of the animals. Ovariectomized control and AI treated mice gained weight constantly throughout the experimental period. E + P (Estrus) and E + P (100/6) group animals showed least weight gain compared to other groups. All values are expressed as mean and for the clarity of the bar-diagram the error bars (representing SD) were removed.

To mimic the postmenopausal breast cancer condition, tumor xenografts were established using aromatase-overexpressing MCF 7 cells in ovariectomized mice. Tumor xenografts in the control ovariectomized mice were relatively fast growing and reached sizes of 500 mm^3^ (8 mm diameter) 12 weeks after transplantation (Figure 2a). Mice treated with anastrozole showed slower tumor growth during the active phase of treatment (designed to replicate the standard 5-year treatment protocol in women), but growth accelerated upon cessation. AI-treated tumors reached 490 mm^3^ after 16 weeks. Treatment of the anastrozole group with continuous E (100 pg/mL) plus P (6 ng/mL) also showed similar effects on the growth of tumor xenografts (500 mm^3^ 16 weeks after tumor transplantation) (Figure 2a). Combination of E (40 pg/mL) and P (6 ng/mL), along with estrous levels of hormones treatment, did not markedly influence tumor xenograft growth compared with ovariectomized controls (Figure 2a). Ovariectomized control and estrous levels of hormone treatment group animals reached ~520 mm^3^ at 13 and 15 weeks respectively, whereas tumor xenografts in E (40 pg/mL)- plus P (6 ng/mL)-treated animals reached sizes of ~550 mm^3^ as early as 12 weeks after transplantation (Figure 2a).

There was a remarkable reduction in the growth of xenografts in animals that received testosterone in addition to E and P. Furthermore, animals that received cyclical menstrual levels of hormone treatment also had reduced tumor growth. These tumors were relatively slow growing and reached sizes of 380 mm^3^ and 420 mm^3^ 19 weeks after xenograft transplantation (Figure 2a). Other slow growing tumor xenografts were observed in the E (100 pg/mL) plus P (6 ng/mL) treatment group, with a latency of 17 weeks to reach a size of 480 mm^3^ (Figure 2a).

### Effect of the aromatase inhibitor and hormones on body weight

Addition of E (100 pg/mL) plus P (6 ng/mL), along with anastrozole treatment, markedly reduced the body weight gain of mice compared with mice treated with AI alone (approximately 16% reduction compared with the AI-treated group) (Figure 2b). This outcome was considered positive because as for humans, weight gain post-ovariectomy results in wide-ranging sequelae in the murine model, with diabetic syndromes and resulting paw and forelimb infection a notable example. Ovariectomized controls and anastrozole-treated animals exhibited maximum body weight gain of all groups, and there were no significant differences between these two groups of mice (Figure 2b). Animals that received estrous levels of E plus P in a cyclic manner had the lowest weight gain. The estrous level of E plus P treatment was effective in reducing the final body mass by 31% compared with ovariectomized control animals (Figure 2b). Ovariectomized mice treated with a combination of E, P, and T had significantly reduced body weights compared with ovariectomized control mice (Figure 2b). Overall, all hormone treatments reduced the weight gain and final body weight of animals compared with ovariectomized controls and anastrozole-treated mice.

### Effect of the aromatase inhibitor and hormones on running wheel performance

**Figure 3:**
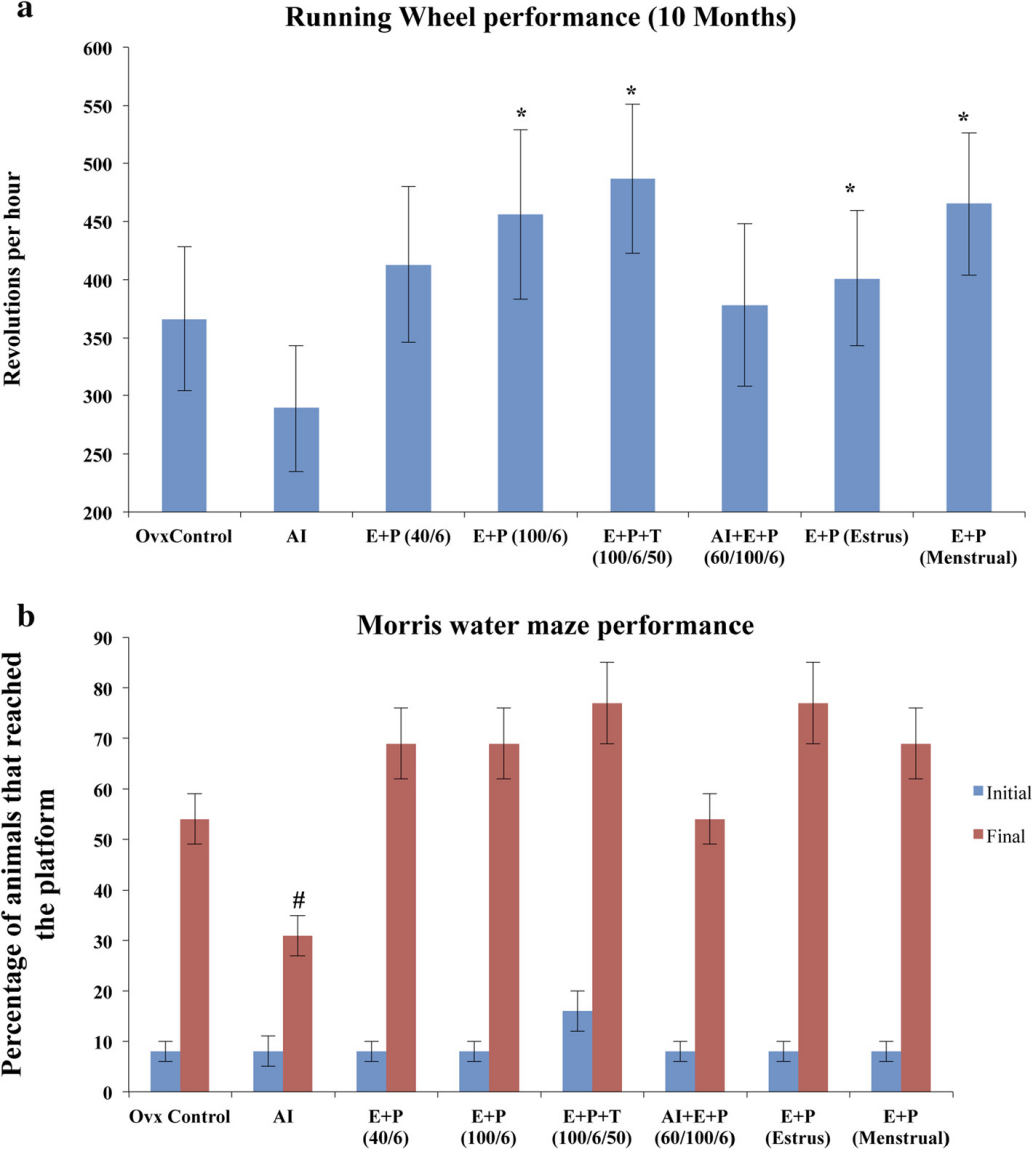
**Effect of hormones on physical activity and cognitive skills. a)** Voluntary running wheel performance shows the physical activity and desire to perform exercise. Revolutions per hour reflect the average physical activity of the animals. AI treatment showed reduced voluntary physical activity whereas E + P + T treatment showed significantly increased desire towards physical activity. E + P (100/6), E + P (Estrus) and E + P (Menstrual) groups also showed significantly improved physical activity compared to AI treated group. **b)** Cognitive function or spatial learning memory of the mice was tested using Morris water maze. All group mice showed similar cognitive ability during initial training. After training, mice in AI treatment group showed least improvement and E + P + T group mice showed better cognitive function than other groups. Since, all 5, 10 and 15-month time points showed similar trend, 10-month time point was taken as a representative result. All values are expressed as Mean ± SD and p ≤ 0.05 was considered statistically significant. *- represents significant difference between AI treatment and other hormone treatments, #- represents significant difference between ovariectomized control and AI treatment.

Because physical activity reflects general health and wellness, we also observed the physical activity of the animals. Running wheel revolutions per hour were monitored as an indicator of physical activity. AI-treated mice had reduced numbers of revolutions per hour on the running wheel compared with ovariectomized control mice indicating that AI treatment induces a comparatively sedentary life style. Supplementation of E + P with AI treatment increased the running wheel performance of mice to control ovariectomized levels at 5 and 10 months (Figure 3a, see Additional file 1: Figure S1a). Animals treated with E (40 pg/mL) plus P (6 ng/mL), E (100 pg/mL) plus P (6 ng/mL), and E plus P plus T significantly increased running wheel activity compared with control ovariectomized mice (Figure 3a). Cyclic treatment with hormones representing estrous and menstrual cycles also improved running wheel performance compared with ovariectomized mice after 5 and 10 months (Figure 3a, see Additional file 1: Figure S1). At the 15-month time point, all groups of animals showed similar performance on the running wheel, but with a slight increase in the steady state level of the hormone treatment groups (See Additional file 1: Figure S1b).

### Water maze

To access spatial learning ability, we used water maze experiments. Initially, all animals showed comparatively equal cognitive behavior with 8 to 16% of animals reaching the platform (Figure 3b). After the trials, mice treated with AI showed the least improvement in spatial learning memory because only 34% of animals succeeded in reaching the platform whereas 54% of ovariectomized controls reached the platform (Figure 3b). This finding may indicate that AI reduces cognition and spatial learning ability. Supplementation with E plus P and AI improved cognition and spatial learning ability to 54% after trials, which showed a positive effective of E and P treatment (Figure 3b). The most effective hormone treatment combination was E plus P plus T in steady state and cyclic hormone treatment mimicking estrous cycles, which showed ~80% improvement in the learning memory of mice (Figure 3b). Further, approximately 70% of animals reached the platform in the E (40 pg/mL) plus P (6 ng/mL), E (100 pg/mL) plus P (6 ng/mL), and menstrual levels of hormone-treated groups (Figure 3b).

### Serum lipids and lipoproteins

**Figure 4:**
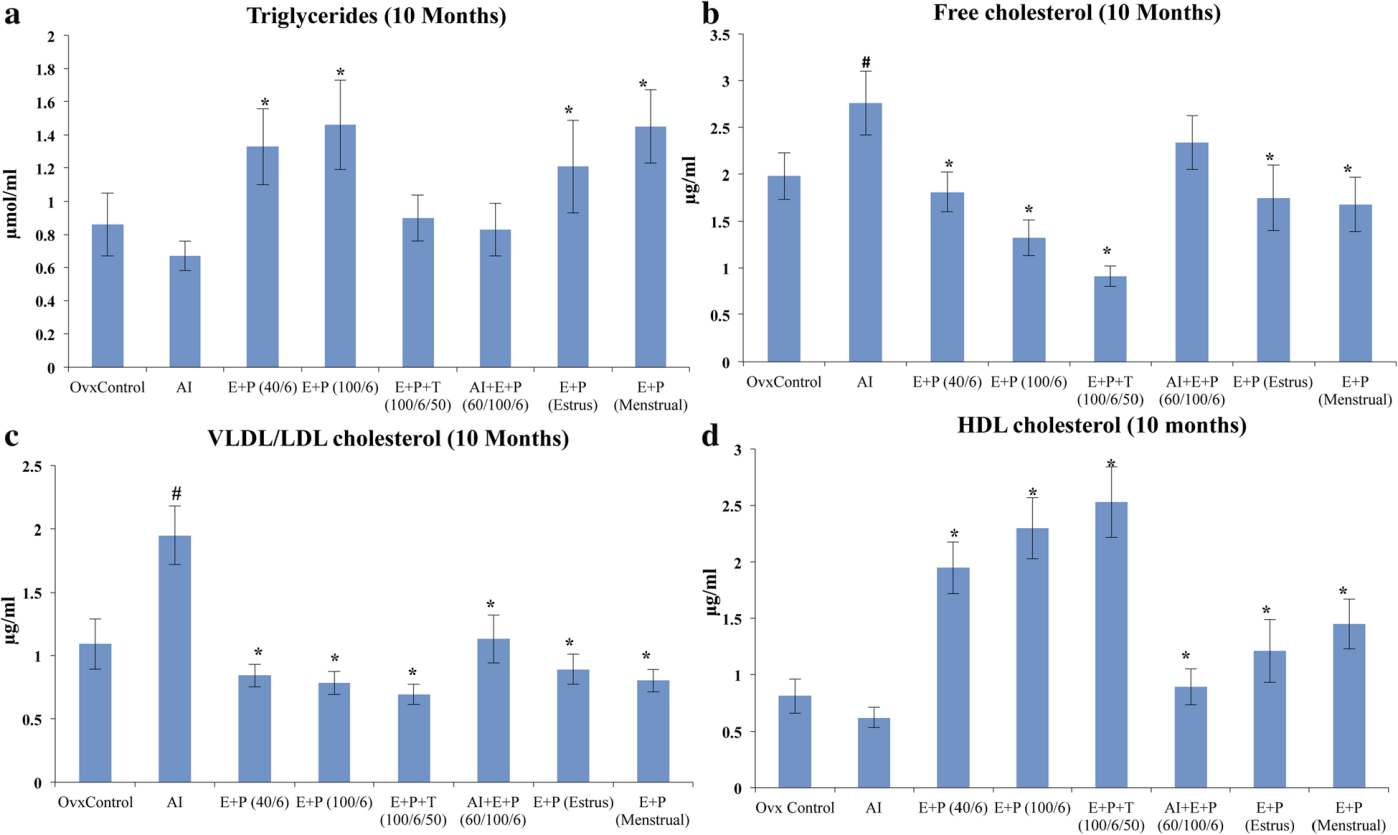
**Effect of hormones on serum lipid and lipoprotein levels. a)** Total serum triglyceride content was significantly increased in E + P (40/6), E + P (100/6), E + P (Menstrual) and E + P (Estrus) groups. **b)** Free cholesterol levels were increased significantly in mice treated with AI compared to ovariectomized control animals. E + P + T treatment showed highly significant reduction compared to ovariectomized control and AI treatment. Other hormone treatments lowered the free serum cholesterol significantly, except AI + E + P. **c)** VLDL/LDL cholesterol levels were significantly increased in AI treatment whereas, the combination of various hormone treatments reduced the VLDL/LDL level significantly. **d)** Serum HDL cholesterol levels were increased significantly in all hormone treated groups compared to AI treatment. Since, all 5, 10 and 15-month time points showed similar trend, 10-month time point was taken as a representative result. All values are expressed as Mean ± SD and p ≤ 0.05 was considered statistically significant. *- represents significant difference between AI treatment and other hormone treatments, #- represents significant difference between ovariectomized control and AI treatment.

Serum lipid and lipoprotein levels were measured to evaluate the effect of AI and hormone treatments on the cardiovascular health of mice. After 5 and 10 months, serum triglyceride levels were decreased in the AI treatment group compared with the ovariectomized control group. Significant increases in triglyceride levels were observed in E (40 pg/mL) plus P (6 ng/mL), E (100 pg/mL) plus P (6 ng/mL), and the estrous and menstrual cycle levels of hormone treatment groups compared with ovariectomized controls (Figure 4a). After 15 months of hormone exposure, the elevated triglyceride levels returned to the levels in ovariectomized controls in E (40 pg/mL) plus P (6 ng/mL), E (100 pg/mL) plus P (6 ng/mL), and estrous and menstrual cycle levels of hormone treatment groups (See Additional file 2: Figure S2b). The E plus P plus T combination treatment showed no difference in triglyceride levels at the 5-, 10-, or 15-month time point (Figure 4a, see Additional file 2: Figure S2a,b). Estimation of free and VLDL/LDL cholesterol levels in serum revealed that AI treatment remarkably increased the levels of these lipids, whereas the addition of E plus P along with AI significantly reduced free cholesterol and VLDL/LDL levels compared with the AI-treated group (Figure 4b,c). The same trend was observed after 5, 10, and 15 months (Figure 4b,c & see Additional file 3: Figure S3 a-d). The highest reduction in cholesterol levels (both free and VLDL/LDL) was observed in E plus P plus T treatment group. Both menstrual and estrous levels had similar effects on reducing the free and VLDL cholesterol in circulation (Figure 4c & see Additional file 3: Figure S3 c,d). HDL, the “good cholesterol”, was significantly reduced in AI-treated animals after 15 months, indicating an increased risk for cardiovascular diseases (See Additional file 4: Figure S4 b). Combination of AI and E plus P treatment returns the levels of HDL to ovariectomized control levels (Figure 4d & see Additional file 4: Figure S4 a). Treatment with E plus P plus T increased serum HDL cholesterol levels compared with ovariectomized controls and could be effective in reducing the risk of cardiovascular diseases (Figure 4d & see Additional file 4: Figure S4 a,b). Both the cyclic levels of hormone treatments increased HDL cholesterol compared with ovariectomized levels after 5 months (See Additional file 4: Figure S4 a). These effects improved and were significant at the 10- and 15-month analyses (Figure 4d & Additional file 4: Figure S4 a,b).

### Serum bone formation markers

**Figure 5:**
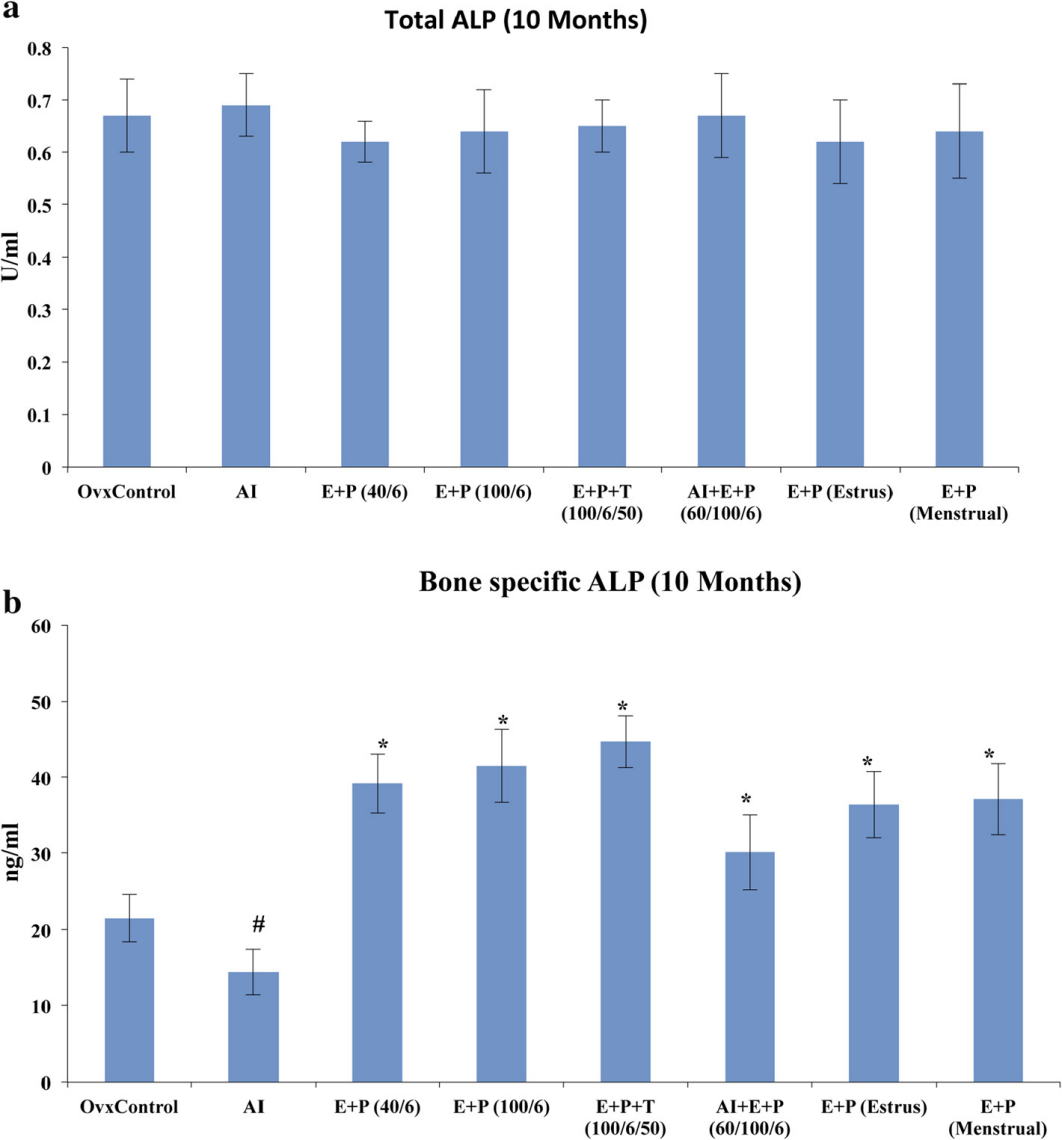
**Effect of hormones on total and bone specific alkaline phosphatase (ALP). a)** There was no significant change in the level of total ALP was observed at 10 months time point. **b)** Bone specific ALP levels were increased significantly in combination of hormone treatments. AI treatment reduced the bone specific ALP levels in the serum showing reduced bone formation process. Since, all 5, 10 and 15-month time points showed similar trend, 10-month time point was taken as a representative result. All values are expressed as Mean ± SD and p ≤ 0.05 was considered statistically significant. *- represents significant difference between AI treatment and other hormone treatments, #- represents significant difference between ovariectomized control and AI treatment.

Analyses of total ALP levels revealed that there was no marked difference in the activity of total ALP in all groups at each time point (Figure 5a & see Additional file 5: Figure S5 a,b). Bone-specific ALP activity assays showed a significant reduction in activity in the AI-treated mice (Figure 5b). Because bone-specific ALP is considered a marker for bone formation, our results indicate that AI treatment negatively influences bone formation. The combination of hormones plus AI treatment significantly increased the activity of bone-specific ALP compared with the AI-treated group (Figure 5b & see Additional file 5: Figure S5 c,d). The steady state [E (40 pg/mL) plus P (6 ng/mL), E (100 pg/mL) plus P (6 ng/mL), and E plus P plus T] and cyclic (estrous and menstrual) hormone treatments showed increased bone-specific ALP activity in serum compared with ovariectomized controls. A similar trend was observed at the 5-, 10-, and 15-month time points (Figure 5b & see Additional file 5: Figure S5 c,d).

## Discussion

Anti-estrogen therapies are currently the standard treatment for estrogen receptor-positive breast cancer recurrence. However, AIs are known to reduce bone mineral density, increasing risk of osteoporosis, and their side effect profile leads to a high discontinuation rate [21].

The current study was performed to investigate hormonal therapeutic regimens that inhibit breast tumor growth without negative effects on cardiovascular and bone formation processes. The ultimate goal of the study was to develop a postmenopausal hormone treatment regimen that blocks the growth of breast cancer while enhancing overall health and quality of life.

The use of hormone replacement therapy for postmenopausal symptoms has been the subject of debate for the past two decades. Studies over that time have revealed a risk-benefit profile that varies by type of hormone, time since menopause, and organ/system in question [24]. In addition, the dose, duration, and mode of administration of hormones are important factors in determining the efficiency and beneficial function of a particular treatment [25,26]. However, current standard of practice considers hormones of any type absolutely contraindicated after hormone-receptor-positive breast cancer, with the assumption being that hormones “throw fuel on the fire” of cancer. This assumption makes intuitive sense, since current treatment is to block remaining estrogens with aromatase inhibitors, the exact opposite.

Yet hormones have myriad effects throughout the body, effects which influence survival and quality of life as much as breast cancer recurrence does or more. We explored a radical hypothesis: Could an optimal choice of hormones lead to improved survival factors and quality of life enough to outweigh any negative effect on tumor recurrence?

In our experiments, we used steroids in their bioidentical form, as these hormones have been shown to possess a more positive risk-benefit profile than synthetic hormones which have been molecularly altered for patentability or oral bioavailability [27-30]. The first question we sought to address was influence on overall health of an optimal hormone regimen. In the landmark Women’s Health Initiative study, a negative risk-benefit profile was seen with oral equine estrogens and oral synthetic medroxyprogesterone acetate (PremPro), a drug combination based on an estrogen formulation first approved in 1942 and which continues to dominate the market in English-speaking countries. We therefore chose estradiol and progesterone delivered non-orally, as is commonly used in southern European countries and increasingly in English-speaking countries as well, based on an extensive literature indicating more favorable global risk-benefit profile [31].Our results show that the right combination of hormone treatments is essential to achieving the desired effect on postmenopausal symptoms and the risks associated with osteoporosis and cardiovascular disease (CVD). E plus P plus T treatment was associated with increased cognition, physical activity, and cardiovascular and bone health in the mouse model, and demonstrates the potential significance of hormone treatment in postmenopausal women. Testosterone is critical to both physical activity and mental health. Testosterone has been shown to be beneficial to cognitive function and memory. It also functions like vasodilator and enhances endothelial functions to improve cardiac health [32,33]. In our study we found that addition of testosterone along with estradiol and progesterone improves the cognitive function, physical activity and cardiac health. In agreement with our study, testosterone therapy has also been shown to reduce breast cancer incidence in postmenopausal women and breast tumor growth in animal models [34-37].

Because estrogen-blocking aromatase inhibitors are the current adjuvant treatment after hormone-sensitive breast cancer, common sense would lead to the assumption that any treatment containing estrogen itself would lead to opposite, highly negative impact on tumor growth. However, this turned out not to be the case. As was the case for general health markers, maximal reduction in tumor growth was achieved by E plus P plus T treatment. In only one group, the lowest-dose E plus P group, did addition of estrogen result in tumor volumes slightly worse than control. Our results thus did not confirm the “throwing fuel on the fire” conception prevalent among clinicians.

Furthermore, the antitumor effect of AI treatment, though notable when compared to control, did not excel when compared to hormone treatment. Treatment with AI had initial antitumor activity, consistent with the results of preclinical studies leading to the approval of AIs. However, three of five hormone treatment regimens provided similar suppression of tumor volume to the AI regimen. And with cessation of the AI treatment phase (chosen to be equivalent to the current clinical standard of care, 5 years), the antitumor effect of AIs diminished, leading to a steepened rise in tumor volume, while the most effective hormone regimens, including E plus P plus T, continued to more effectively suppress tumor volume.

A frequent criticism of studies in a mouse model is that they may have limited utility in predicting eventual clinical outcomes. Anticipating this objection, we designed our study to exactly mimic conditions such as dose and length of treatment used in pre-approval studies of anastrozole (Arimidex®), a leading aromatase inhibitor currently on the market. These murine-model results were seen to accurately predict outcomes later seen in large clinical trials such as the ATAC (Arimidex, Tamoxifen, Alone or in Combination) trial and were part of the basis for approval of anastrozole [38,39].

Although an E plus P plus T regimen performed better in our study than AIs (the current standard of care) on measures of both tumor growth and general health, considerable momentum, as well as market forces, works against a reversal in treatment practice from hormone inhibitors to hormones. We therefore sought to determine whether addition of optimal hormones could improve quality of life and general health indicators when added to—instead of substituting for—AI treatment, without worsening tumor outcomes. Our results indicated the viability of this approach. When added to AIs, estradiol and progesterone significantly improved the general health of the animals as measured by cardiac and bone health markers (although positive impact of hormones on cardiac and bone health markers was not as marked when added to AIs as when used alone), without promoting breast tumor growth. We discuss possible explanations for this seeming paradox—improved general health but lack of tumor stimulation—below.

Aromatase classically converts C19 steroids (androgens) to C18 steroids (estrogens) with the addition of a hydroxyl group. Because it has a wide range of substrate specificity, it accepts DHEA as a substrate and converts it to estrogens [40,41]. Estrogen, which is predominantly produced in postmenopausal women by the aromatization of DHEA, selectively activates ERα [42,43]. Aromatase has high expression in breast tumor cells and the surrounding stroma in postmenopausal women [44,45]. AIs inhibit aromatase and reduce the conversion of androgens to estrogens in postmenopausal breast cancer patients.

However, this inhibition is not reliably effective long-term because many patients develop resistance to AI treatment [46-48]. Furthermore, inhibiting aromatase in tissues outside the breast is associated with a variety of negative sequelae in joints, bone, and other tissues [49-53]. Our data indicate that an optimal dose of estradiol and progesterone can overcome effects on bone, cardiovascular, and cognitive health. Furthermore, the addition of testosterone along with estradiol and progesterone enhances the beneficial effects.

Large observational studies suggest estrogens have a cardioprotective effect [13,54,55]. Abnormal serum lipid levels have been associated with an increased risk for CVDs [13,54]. Low HDL and high LDL levels in serum are mainly attributed to an increased risk of CVDs. Several clinical and experimental studies indicate that estradiol treatment is beneficial to the heart by reducing LDL levels and increasing HDL levels in circulation [56-58]. Based on epidemiological studies, CVDs are prevalent in postmenopausal women, and serum concentrations of estrogen are inversely associated with CVD risk [59]. Estrogen replacement therapy initiated within 5 years after menopause has a beneficial effect on cardiovascular risk factors, but not if the therapy is begun later [60]. Natural hormone 17-b estradiol was more effective in reducing CVD risk factors than conjugated equine estrogens, and it is also affected by an oral or transdermal route of delivery [61]. Taking all of these factors into account, the cellular mechanism of estradiol-induced cardioprotection involves the contribution of several factors including time of administration, type of hormone administered, and mode of administration. Our data demonstrate that administration of hormones immediately after ovariectomy results in improved cardiac health, in agreement with data from clinical studies.

The role of progesterone versus synthetic progestins in cardiovascular health is the subject of much debate. The WHI study demonstrated increased atherosclerosis upon the use of synthetic progestins [13,62,63]. In a long-term randomized study that accessed and compared the effects of synthetic progestins and progesterone on serum lipids, synthetic progestins negatively influenced the beneficial effect of estrogens by lowering serum HDL levels [55]. Progesterone, in contrast, has been shown to support the cardioprotective actions of estrogen in several other studies [64-70]. Our results indicate that estrogen and progesterone improve the serum lipid profile and reduce the risk of CVDs in a postmenopausal breast cancer mouse model.

Osteoporosis is a major concern in postmenopausal women. Several studies have shown that estradiol increases bone formation and prevents osteoporosis [71,72]. Similarly, depletion of estrogen resulted in osteoporosis, supporting the notion that estrogens are important for bone formation [73,74]. Clinical studies have indicated that progesterone treatment helps maintain bone mass [75-77]. Progesterone supports bone formation by preventing glucocorticoid-induced bone loss [54]. Several animal and human studies have demonstrated progesterone’s positive effect on bone formation as well as inhibition of bone resorption [76-78]. Studies evaluating estrogen and progesterone supplementation suggest estrogen and progesterone have distinct but complementary roles in bone maintenance [75-77,79]. The addition of testosterone positively influences bone mass by preventing urinary calcium loss. Our findings demonstrate that the addition of hormones along with AI treatment is beneficial for bone health in postmenopausal women.

Our data on physical activity, cognition, and spatial learning clearly demonstrate the importance of hormones in addition to AIs for breast cancer treatment. Cyclical administration of hormones appears to have a slightly better effect versus administration of steady levels of hormones. It is interesting that the addition of testosterone has a significant positive impact on all aspects that were studied in this investigation.

## Conclusions

In summary, our results indicate that the use of appropriate combinations of natural hormones along with, or instead of, classical breast cancer treatments is beneficial against postmenopausal symptoms and improves cardiac and osteoporotic health in the mouse model. The natural hormone combinations tested in this study provide evidence for a better alternative to standard aromatase inhibitor treatment following breast cancer in women.

## Abbreviations

AI: Aromatase inhibitor; ALP: Alkaline phosphatase; ATAC: Arimidex; Tamoxifen: Alone or in Combination; CVD: Cardiovascular disease; DHEA: Dehydroepiandrosterone; E: Estrogen; HDL: High density lipoprotein; HRT: Hormone replacement therapy; LDL: Low density lipoprotein; P: Progesterone; T: Testosterone; VLDL: Very low density lipoprotein; WHI: Women’s health initiative.

## Competing interests

The authors declare that they have no competing interests.

## Authors’ contributions

RL designed the study, carried out the experiments, supervised the project and prepared the manuscript. AA carried out the experiments, collected and analyzed the data. EL conceived of the study, participated in study design, and was involved in manuscript preparation. All authors read and approved the final manuscript.

## Supplementary Material

Additional file 1: Figure S1 Effect of hormones on physical activity. Running wheel performances of mice at 5 and 15-month time points. All values are expressed as Mean ± SD and p ≤ 0.05 was considered statistically significant. *- represents significant difference between AI treatment and other hormone treatments, #- represents significant difference between ovariectomized control and AI treatment. Click here for file

Additional file 2: Figure S2 Effect of hormone treatments on serum triglycerides. Serum triglycerides levels at 5-month time point showed similar trends compared to 10-month time point. At 15^th^ month the level of triglycerides were similar in all the groups. All values are expressed as Mean ± SD and p ≤ 0.05 was considered statistically significant. *- represents significant difference between AI treatment and other hormone treatments, #- represents significant difference between ovariectomized control and AI treatment. Click here for file

Additional file 3: Figure S3 Effect of hormone treatments on serum free cholesterol and VLDL/LDL cholesterol. The level of free cholesterol was reduced in the E + P (100/6), E + P + T, E + P (Menstrual) and E + P (Estrus) groups in both 5 and 15 month time points. *- represents significant difference between AI treatment and other hormone treatments, #- represents significant difference between ovariectomized control and AI treatment. Click here for file

Additional file 4: Figure S4 Effect of hormone treatments on serum HDL cholesterol. The level of free cholesterol was reduced in the AI treatment but increased in other hormone treatments in both 5 and 15-month time points. *- represents significant difference between AI treatment and other hormone treatments, #- represents significant difference between ovariectomized control and AI treatment. Click here for file

Additional file 5: Figure S5 Effect of hormones on total and bone specific ALP. Levels of ALP in all the groups were similar in all the time points. Bone specific ALP levels were increased in all the hormone treated groups. *- represents significant difference between AI treatment and other hormone treatments, #- represents significant difference between ovariectomized control and AI treatment. Click here for file
