# Supplementary figures and images for: The role of hormones and aromatase inhibitors on breast tumor growth and general health in a postmenopausal mouse model
Source: Reprod Biol Endocrinol. 2014 Jul 15;12:66. doi: 10.1186/1477-7827-12-66 (PMC4110932; doi:10.1186/1477-7827-12-66)

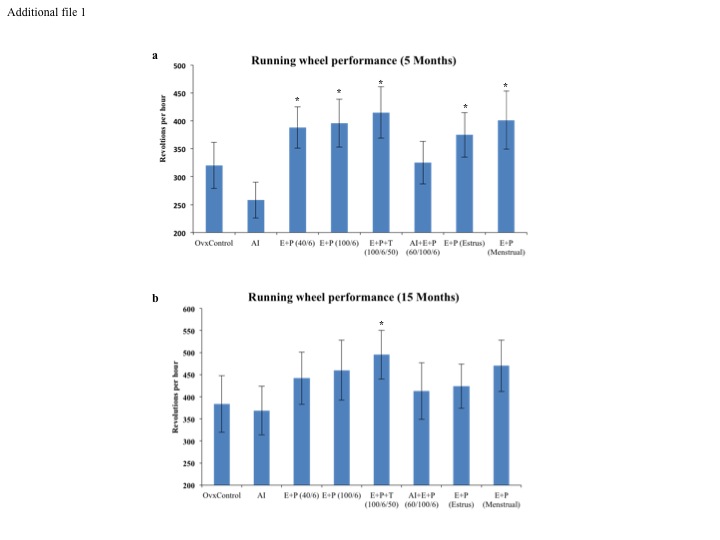

Supplement: Additional file 1: Figure S1 — Effect of hormones on physical activity. Running wheel performances of mice at 5 and 15-month time points. All values are expressed as Mean ± SD and p ≤ 0.05 was considered statistically significant. *- represents significant difference between AI treatment and other hormone treatments, #- represents significant difference between ovariectomized control and AI treatment. [file 1477-7827-12-66-S1.jpeg]

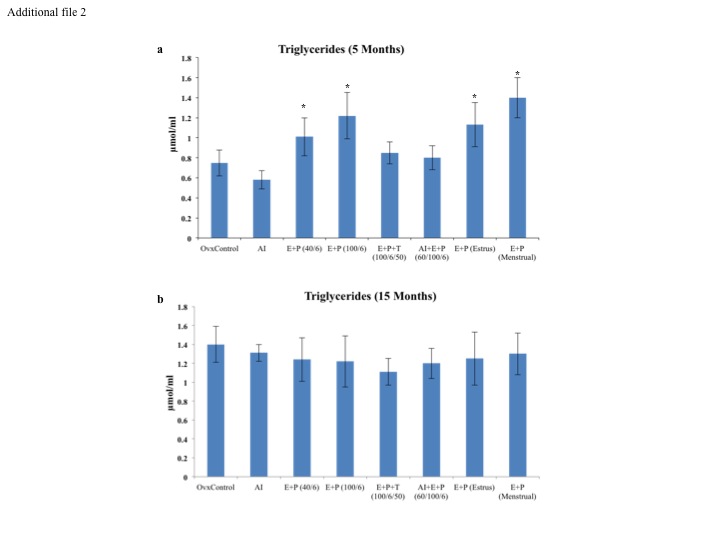

Supplement: Additional file 2: Figure S2 — Effect of hormone treatments on serum triglycerides. Serum triglycerides levels at 5-month time point showed similar trends compared to 10-month time point. At 15th month the level of triglycerides were similar in all the groups. All values are expressed as Mean ± SD and p ≤ 0.05 was considered statistically significant. *- represents significant difference between AI treatment and other hormone treatments, #- represents significant difference between ovariectomized control and AI treatment. [file 1477-7827-12-66-S2.jpeg]

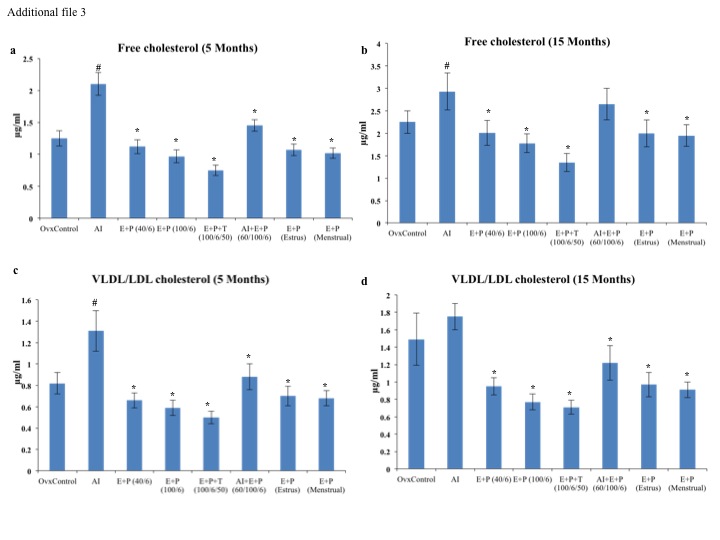

Supplement: Additional file 3: Figure S3 — Effect of hormone treatments on serum free cholesterol and VLDL/LDL cholesterol. The level of free cholesterol was reduced in the E + P (100/6), E + P + T, E + P (Menstrual) and E + P (Estrus) groups in both 5 and 15 month time points. *- represents significant difference between AI treatment and other hormone treatments, #- represents significant difference between ovariectomized control and AI treatment. [file 1477-7827-12-66-S3.jpeg]

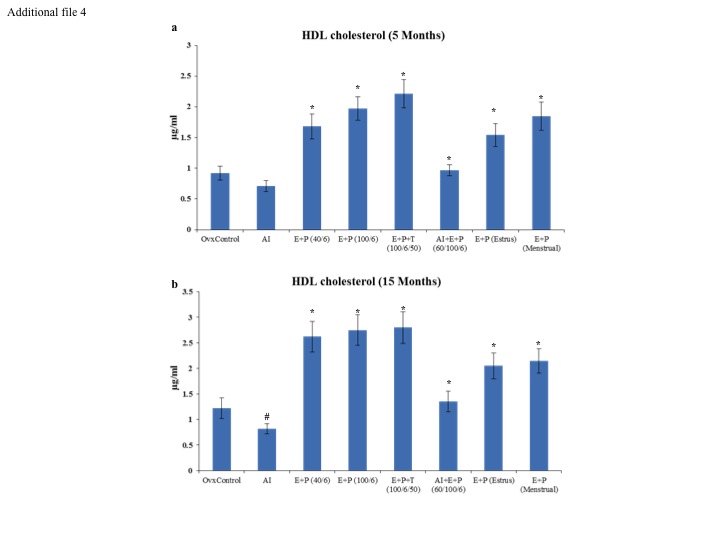

Supplement: Additional file 4: Figure S4 — Effect of hormone treatments on serum HDL cholesterol. The level of free cholesterol was reduced in the AI treatment but increased in other hormone treatments in both 5 and 15-month time points. *- represents significant difference between AI treatment and other hormone treatments, #- represents significant difference between ovariectomized control and AI treatment. [file 1477-7827-12-66-S4.jpeg]

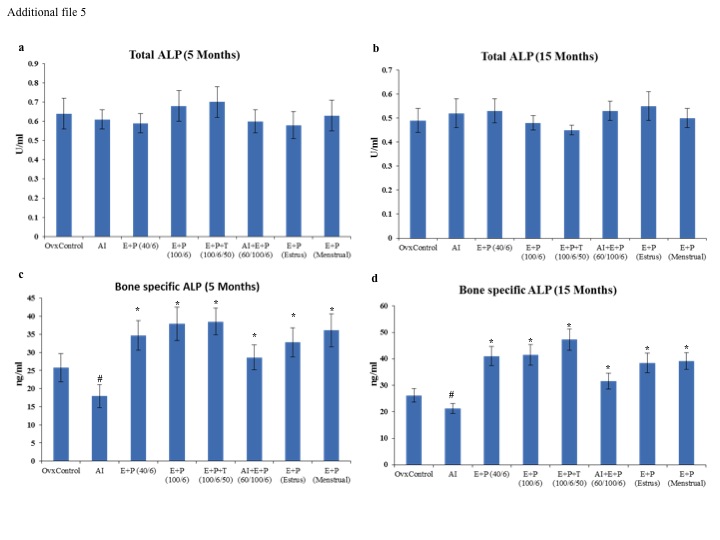

Supplement: Additional file 5: Figure S5 — Effect of hormones on total and bone specific ALP. Levels of ALP in all the groups were similar in all the time points. Bone specific ALP levels were increased in all the hormone treated groups. *- represents significant difference between AI treatment and other hormone treatments, #- represents significant difference between ovariectomized control and AI treatment. [file 1477-7827-12-66-S5.jpeg]
